# Structure of the {U_13_} polyoxo cluster U_13_O_8_Cl_*x*_(MeO)_38–*x*_ (*x* = 2.3, MeO = methoxide)

**DOI:** 10.1107/S2056989021007623

**Published:** 2021-07-30

**Authors:** Sebastian Fichter, Thomas Radoske, Atsushi Ikeda-Ohno

**Affiliations:** a Helmholtz-Zentrum Dresden-Rossendorf (HZDR), Institute of Resource Ecology, Bautzner Landstrasse 400, 01328 Dresden, Germany; b Collaborative Laboratories for Advanced Decommissioning Science (CLADS), Japan Atomic Energy Agency (JAEA), 2-4 Shirakata, Tokai-mura, Naka-gun, 319-1195 Ibaraki-ken, Japan

**Keywords:** polyoxo cluster, *f*-block elements, actinides, uranium, tetra­valent, crystal structure

## Abstract

The structure of a new type of polyoxo cluster complex that contains thirteen uranium atoms, {U_13_}, is reported. The complex crystallized from methanol containing tetra­valent uranium (U^IV^) with a basic organic ligand, and was characterized as di­chloridoocta­cosa-μ_2_-methano­lato-octa­kis­(methano­lato)octa-μ_4_-oxido-trideca­uranium, [U_13_(CH_3_O)_35.7_Cl_2.3_O_8_] or [U_13_(μ_4_-O_oxo_)_8_Cl_*x*_(MeO)_38–*x*_] (*x* = 2.3, MeO = methoxide) (**I**), by single-crystal X-ray diffraction. The characterized {U_13_} polyoxo cluster complex (**I**) possesses a single cubic uranium polyhedron at the centre of the cluster core. To the best of our knowledge, this is the very first example of a polyoxo actinide complex that bears a single cubic polyhedron in its structure. The cubic polyhedron in **I** is well comparable in shape with those in bulk UO_2_. The U—O bonds in the cubic polyhedron of **I** are, however, significantly shorter than those not only in bulk UO_2_ but also in another analogue in the {U_38_} cluster. This shortening of U—O bonds, together with BVS calculations and the overall negative charge (2−) of **I**, suggests that the central uranium atom in **I**, which forms the single cubic coordination polyhedron, is presumably oxidized to the penta­valent state (U^V^) from the original tetra­valent state (U^IV^). Complex **I** is, hence, the first example of a polyoxo cluster possessing a single cubic coordination polyhedron of U^V^.

## Abstract 2

A new type of uranium polyoxo cluster complex consisting of thirteen uranium atoms, [U_13_(μ_4_-O_oxo_)_8_Cl_*x*_(MeO)_38-*x*_] (*x* = 2.3, MeO: methoxide), was synthesized and structurally characterized by single crystal X-ray diffraction.

## Chemical context   

Hydrolysis is one of the most fundamental reactions in aqueous chemistry. The strong hydrolysis of highly charged metal cations (*M^+n^*) induces olation (to form hydroxo-bridging: *M*–OH–*M*) and oxolation (to form oxo-bridging: *M*–O–*M*), which eventually results in the formation of hydroxo/oxo-bridged oligomer and cluster complexes in an aqueous solution (Henry *et al.*, 1992 ▸). Amongst the hydroxo/oxo-based oligomer/cluster complexes of metal cations, the polyoxo cluster complexes of *f*-block elements (*i.e*. lanthanides and actinides) have been extensively investigated over the last few decades, not only for their engineering applications and environmental impact associated with nuclear industry, but also for the fundamental chemical science of *f*-block elements (Knope & Soderholm, 2013 ▸; Qiu & Burns, 2013 ▸). As a discrete polyoxo cluster complex (*i.e*. not a chain- or wheel-shaped cluster) of *f*-block elements, the largest cluster complex reported thus far is the cluster containing 100 metal cations ({*M*_100_}) (Russell-Webster *et al.*, 2021 ▸), within which a large variety of nuclearity was reported. Based on this background, the present work contributes to further development of the polyoxo cluster chemistry of *f*-block metals by reporting a new member of the polyoxo cluster family of tetra­valent uranium (U^IV^) that contains thirteen metal centres: {U_13_}. 
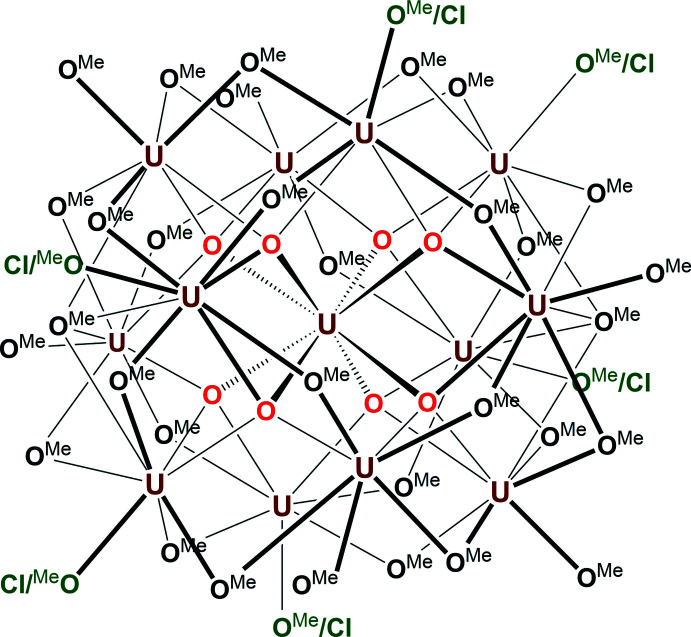


## Structural commentary   

**Figure 1:**
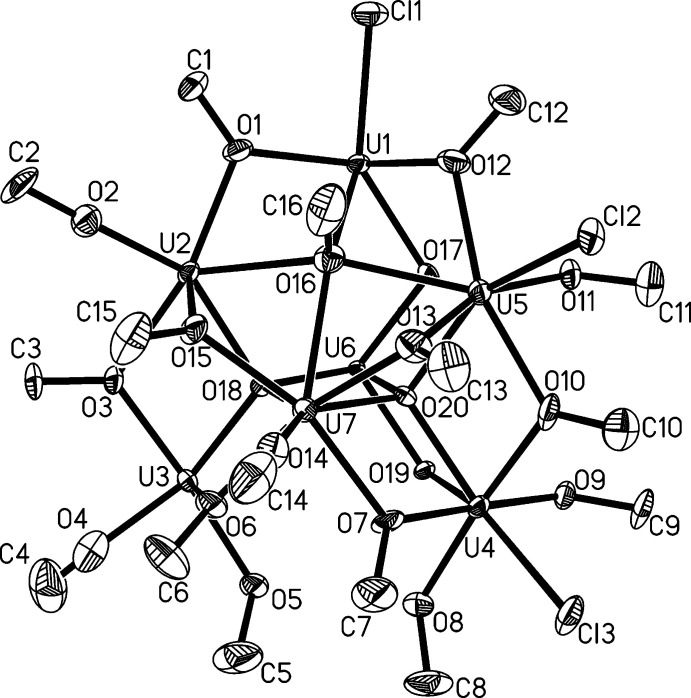
Mol­ecular structure of the asymmetric unit of the {U_13_} cluster **I**. Ellipsoids are shown at the 50% probability level. H atoms are omitted for clarity, as are the disordered methoxide ligands (only the chlorides that share their locations are shown).

**Figure 2:**
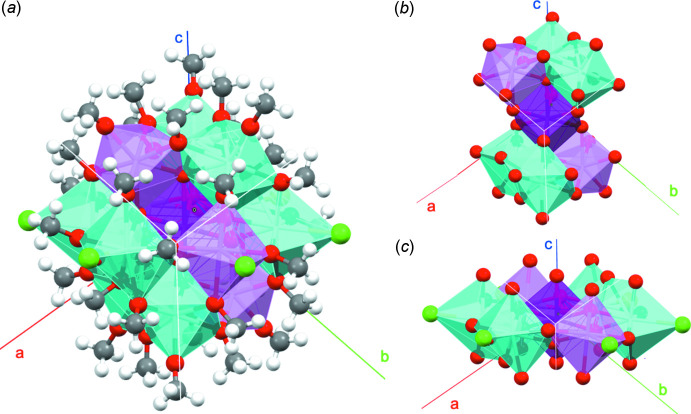
Mol­ecular structure of the {U_13_} cluster **I**. Uranium atoms are illustrated with coloured polyhedra. The structure is drawn as [U_13_(μ_4_-O_oxo_)_8_Cl_6_(MeO)_32_] in order to omit the disorder between chloride and methoxide anions for clarity. Colour code: hydrogen, white; carbon, black; oxygen, red; chlorine, light green. Hydrogen and carbon atoms are also omitted for clarity in (*b*) and (*c*).

The best refinement for the SC-XRD data of the dark-black crystals resulted in the chemical formula C_35.7_H_107.1_Cl_2.3_O_43.7_U_13_, which corresponds to the mol­ecular formula [U_13_(μ_4_-O_oxo_)_8_(μ_4_-O_MeO_)_2_(μ_2_-O_MeO_)_24_Cl_2.3_(O_MeO_)_9.7_] (**I**). The mol­ecular structure of **I** (*i.e.* the {U_13_} cluster) contains seven distinct crystallographically independent uranium centres (U1–U7), which are bridged by eight μ_4_-O_oxo_, two μ_4_-O_MeO_, and twenty-four μ_2_-O_MeO_ oxygen donors to form the {U_13_} core. The exterior of the {U_13_} core is further decorated with monodentate chloro and methoxide (MeO) ligands to complete the uranium centres’ coordination spheres in terminal positions, eventually forming the {U_13_} cluster compound (**I**) (Figs. 1 ▸ and 2 ▸ *a*). In the crystal structure, there is some disorder between the chloro and methoxide ligands at the terminal positions (*i.e*. Cl1–Cl3). This means that partial chloro ligands and partial methoxide groups occupy the same coordination sites in the average structure and that they can be found on either of three out of the seven refined uranium centres of the asymmetric unit. Given this fact, it is more appropriate to describe the mol­ecular formula of **I** as [U_13_(μ_4_-O_oxo_)_8_Cl_*x*_(MeO)_38-*x*_], where *x* was determined to be 2.3 by SC-XRD. The uranium centres in **I** are mostly eightfold coordin­ated, whilst only U3 and U4 are sevenfold coordinated (pink polyhedra in Fig. 2 ▸). One uranium centre (U6), which is positioned at the centre of the {U_13_} core unit, forms a nearly ideal cubic polyhedron (dark-purple polyhedra in Fig. 2 ▸), whilst the rest of the eightfold coordinated uranium centres (U1, U2, U5 and U7) define distorted square-anti­prismatic polyhedra (green polyhedra in Fig. 2 ▸). The central cubic uranium polyhedron (U6) is sandwiched with two {U_3_} sub­units (pink and green polyhedra in Fig. 2 ▸ *b*) along the *c-*axis direction, and it is further surrounded by a {U_6_} ring (pink and green polyhedra in Fig. 2 ▸ *c*). Hence, one cubic uranium polyhedron, two {U_3_} subunits, and one {U_6_} ring assemble to the {U_13_} core [*i.e*. 1 + (2 × 3) + 6 = 13]. The sevenfold-coordinated uranium centres (U3 and U4, pink polyhedra in Fig. 2 ▸), from a different perspective, form the corners of a square around the central U6, the edges of which are open to allow for a direct view of the central cubic uranium centre as in Fig. 2 ▸ *a*. The {U_13_} core unit is surrounded by chloro and methoxide ligands to stabilize the {U_13_} cluster as a discrete mol­ecule. The structural arrangement of the {U_13_} cluster is well comparable with that of the reported {Ce_13_} cluster: Ce_13_O_8_[(OCH_2_CH_2_)_2_N((C_6_H_5_)]_18_ (Yuan *et al.*, 2017 ▸), in which a single cubic polyhedron of the central cerium centre is surrounded by two {Ce_3_} and one {Ce_6_} ring subunits with distorted square-anti­prismatic polyhedra.

When assuming the formal oxidation numbers of +4 for U^IV^, −2 for oxo groups, and −1 for chloride ions and methoxides, the overall charge of the mol­ecule [U_13_(μ_4_-O_oxo_)_8_Cl_*x*_(MeO)_38-*x*_] is calculated to be −2, which is not neutral. Such an unbalanced charge is often observed for the polyoxo cluster complexes of *f*-block elements (*e.g*. Takao *et al.*, 2009 ▸; Falaise *et al.*, 2013*c* ▸). In fact, the bond-valence-sum (BVS) calculation (Brown, 1978 ▸) [*R* ^0^_U–O_ = 2.10 (Gagné & Hawthorne, 2015 ▸) and *R* ^0^_U–Cl_ = 2.47 (Zachariasen, 1978 ▸)] suggests 4.11 valence units (v.u.) for the average charge of thirteen uranium atoms in **I** (Table 1 ▸), which is higher than the formal charge of U^IV^ (*i.e*. > +4). The results of BVS calculations further indicate that the BVS charge of the U6 atom, which is the central uranium atom in the {U_13_} cluster (dark-purple polyhedra in Fig. 2 ▸), is comparable to the penta­valent state (5.16 v.u.), whilst the rest of the uranium atoms (U1–U5 and U7) exhibit BVS charges close to 4 v.u. (*i.e*. the original tetra­valent state) (Table 1 ▸). Hence, the central uranium atom U6 in **I** is presumably oxidized to U^V^, partly compensating the negative charge of the oxo, chloride and methoxide anions to neutralize the whole mol­ecule. Similar partial oxidation of U^IV^ was also presumed for a {U_38_} polyoxo cluster (Falaise *et al.*, 2013*c* ▸). Another possible charge compensation to keep the neutrality of **I** is the replacement of oxo (−2) by hydroxo ligands (−1). That is, the bridging oxo ions (or methoxide groups) in **I** could be partly protonated, which was also proposed in the {U_38_} polyoxo cluster (Falaise *et al.*, 2013*c* ▸). Hence, a partial protonation and the oxidation of U^IV^ to U^V^ presumably compensate the negative charges of oxo, chloride and methoxide anions and result in a neutral mol­ecule of **I**.

**Figure 3:**
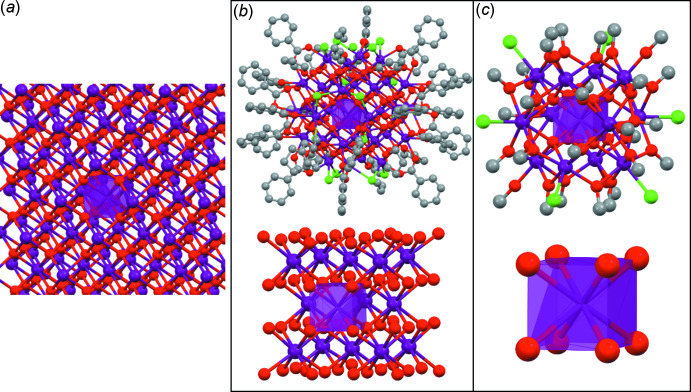
Cubic coordination polyhedra of uranium (dark purple polyhedra) in different compounds: (*a*) bulk UO_2_ (Cooper, 1982 ▸), (*b*) the {U_38_} cluster (Falaise *et al.*, 2013*c* ▸), and (*c*) the {U_13_} cluster (**I**). The structure of **I** is drawn as [U_13_(μ_4_-O_oxo_)_8_Cl_6_(MeO)_32_] in order to omit the disorder between chloride and methoxide anions for clarity. Colour code: carbon, black; oxygen, red; chlorine, light green; uranium, dark purple. Hydrogen atoms are omitted for clarity in (*b*) and (*c*).

The structures of polyoxo clusters of metal cations are often compared with those of their corresponding oxide compounds, as the polyoxo clusters can be potential precursors, which evolve into bulk oxides (Ikeda-Ohno *et al.*, 2013 ▸). In the case of U^IV^ polyoxo clusters, the corresponding oxide is uranium dioxide (UO_2_). The coordination polyhedron of uranium in UO_2_ is cubic, as shown in Fig. 3 ▸ *a* (dark-purple polyhedron). Amongst the reported polyoxo oligomer and cluster complexes of U^IV^ [*i.e.* dimers (Le Borgne *et al.*, 2002 ▸; Salmon *et al.*, 2006 ▸; Schmidt *et al.*, 2014 ▸), trimers (Berthet *et al.*, 1993 ▸; Duval *et al.*, 2015 ▸; Lin *et al.*, 2018 ▸), tetra­mer (Falaise *et al.*, 2013*a* ▸), hexa­mers (Mokry *et al.*, 1996 ▸; Takao *et al.*, 2009 ▸; Mougel *et al.*, 2010 ▸; Falaise *et al.*, 2013*b* ▸), octa­mer (Salmon *et al.*, 2004 ▸), deca­mer (Biswas *et al.*, 2011 ▸), 14-mer (Dufaye *et al.*, 2019 ▸), 16-mer (Biswas *et al.*, 2011 ▸), and 38-mers (Falaise *et al.*, 2013*c* ▸; Martin *et al.*, 2018 ▸)], only the 38-mers {U_38_} contain cubic coordination polyhedra of uranium, which are comparable to those in bulk UO_2_. That is, the {U_14_} core unit in the {U_38_} cluster consists of fourteen cubic uranium polyhedra, corresponding to a small fraction of face-centred cubic UO_2_ (Fig. 3 ▸ *a* and *b*). The central uranium (U6) in the {U_13_} cluster (**I**) (dark-purple polyhedron in Fig. 3 ▸ *c*) also defines a cubic coordination polyhedron. The cubic polyhedron in the {U_13_} cluster is, however, not surrounded by other cubic uranium polyhedra to evolve into a fraction of *fcc*-based UO_2_ structure. Hence, the {U_13_} cluster contains the smallest unit of cubic uranium polyhedron that is comparable to that in UO_2_. Geometrical parameters of the cubic uranium polyhedra in bulk UO_2_, the {U_38_} cluster, and the {U_13_} cluster are summarized in Table 2 ▸. The average O—U—O angle in the cubic uranium polyhedra is 70.5° for all three species, indicating that the shape of the uranium polyhedron is an ideal cube even in the polyoxo clusters. The average U—O distance, however, shortens with decreasing size of the polyhedral cluster. That is, the average U—O distance shortens from 2.368 to 2.357 Å when the size of the polyhedral cluster reduces from bulk UO_2_ (infinite cluster) to {U_14_} (sub-unit in the {U_38_} cluster). The U—O distance further shortens to 2.267 Å, which is ∼5% shorter compared with that in bulk UO_2_, in the case of the single cubic uranium polyhedron in the {U_13_} cluster. This 5% shortening of the U—O distance in the single cubic uranium polyhedron of **I** is rather remarkable. As a matter of fact, such drastic shortening of *M*—O distances is not observed in the {Ce_13_} cluster (Yuan *et al.*, 2017 ▸), the chemical analogue of the {U_13_} cluster. That is, the Ce—O distances (average: 2.35 Å) in the central cubic polyhedron of the {Ce_13_} cluster are well comparable with those in bulk CeO_2_ (2.34 Å) (Wyckoff, 1963 ▸). Given these facts, it is reasonable to consider that the oxidation state of the uranium ion in the single cubic polyhedron (*i.e*. U6) is higher than the original tetra­valent state of U^IV^, strengthening (and thereby shortening) the U—O bonds. This also supports the BVS results suggesting a penta­valent state for uranium centre U6 (U^V^). Hence, the central uranium polyhedron in **I** (dark-purple polyhedra in Fig. 2 ▸) should be considered an exceptionally rare example of a U^V^ polyhedron with a cubic structure, which is comparable with the cubic U^IV^ polyhedron as in UO_2_.

Amongst the polyoxo/hydroxo metal clusters comprising thirteen metal centres ({*M*_13_}), the Keggin-type {Al_13_} cluster is probably the most famous complex of this type (Johansson *et al.*, 1960 ▸; Rowsell & Nazar, 2000 ▸). The {Al_13_} cluster consists of a central aluminium tetra­hedron [Al(O)_4_] that links four trimeric octa­hedra [Al(O)_6_], forming the cluster unit with a diameter of ∼10 Å (assuming a sphere). The {U_13_} cluster characterized in this study is composed of one central uranium polyhedron [cubic-U(O)_8_] surrounded by twelve exterior uranium polyhedra (*i.e*. two {U_3_} subunits and one {U_6_} ring), forming an ellipsoidal cluster *ca* 7 Å wide and 10 Å high. Although {Al_13_} and {U_13_} have the same nuclearity of thirteen, the constituent polyhedra and the framework of the resultant {*M*_13_} cluster differ significantly between {Al_13_} and {U_13_}, reflecting the differences of the metal centres (*i.e*. Al^III^ *vs* U^IV^, as well as their coordination properties). Additionally, given the number of atoms in the polyoxo {*M*_13_} unit (*i.e*. Al_13_O_40_ for {Al_13_} and U_13_O_46_ for {U_13_} assuming 100% occupancy of MeO at all terminal positions) and the dimensions of the cluster, the {U_13_} unit is apparently denser than the {Al_13_} one. Therefore, despite having the same nuclearity of thirteen, {Al_13_} and {U_13_} are actually not well comparable in terms of structure and coordination chemistry.

## Supra­molecular features   

**Figure 4:**
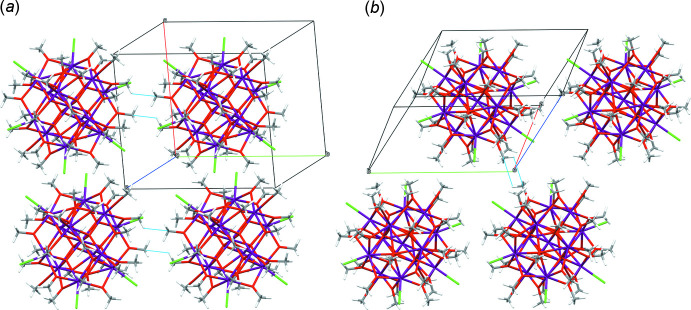
Packing diagrams of **I**. Light-blue lines indicate the inter­molecular short contacts [(*a*) SC1 and (*b*) SC2] between hydrogens and carbons of adjacent methoxide groups. The structure is drawn as [U_13_(μ_4_-O_oxo_)_8_Cl_6_(MeO)_32_] in order to omit the disorder between chloride and methoxide anions for clarity reasons.

Compound **I** crystallizes in the space group *P* 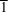 . The chemically analogous {U_38_} cluster crystallizes in a more symmetric crystal system in tetra­gonal setting (*I*4/*m*) (Falaise *et al.*, 2013*c* ▸). This symmetrical difference in crystal structure between the {U_13_} and {U_38_} clusters may stem from the symmetrical difference in their original mol­ecular structures. That is, as shown in Fig. 2 ▸ *a*, the mol­ecular structure of **I** (*i.e*. the {U_13_} cluster) is slightly oval along the *c* axis, whilst the shape of the {U_38_} cluster mol­ecule is rather close to a sphere (Falaise *et al.*, 2013*c* ▸). In the crystal structure of **I**, there are two sets of inter­molecular short contacts that help the mol­ecules to assemble into the crystal structure. These inter­molecular short contacts are indicated with light blue lines in Fig. 4 ▸. One set of inter­molecular short contact (SC1) is found between a hydrogen atom of one bridging methoxide group and a carbon atom of another bridging methoxide group from the adjacent mol­ecule [C7—H8*A* ^i^ = 2.87 Å; symmetry code: (i) 1 − *x*, 2 − *y*, 1 − *z*, Fig. 4 ▸ *a*]. There are two such (bi-directional) SC1 between adjacent mol­ecules, facilitating the mol­ecules being lined up along the *b-*axis direction. A similar C—H inter­molecular short contact (SC2) is formed between a bridging methoxide group and its analogue in an adjacent mol­ecule [C10—H10*B* ^ii^ = 2.89 Å; symmetry code: (ii) 2 − *x*, 2 − *y*, 2 − *z*, Fig. 4 ▸ *b*]. Again pairs of this H—C short contact are found between adjacent bridging methoxide mol­ecules (Fig. 4 ▸ *b*), supporting the assembly of mol­ecules of **I** more or less along a diagonal through the cell’s origin. The engaged bridging methoxide groups are not affected by the disorders between chloride and methoxide groups. These two types of inter­molecular short contacts are, hence, presumably key to assembling the mol­ecules for crystallization. This renders the exterior methoxide groups of **I**, therefore, important not only for stabilizing the discrete {U_13_} core, but also for supporting the assembly and crystallization of the {U_13_} mol­ecules and the stability of the resulting crystal lattice.

## Synthesis and crystallization   

*Caution!* Uranium isotopes (^235^U and ^238^U) are long-lived α-emitters with half-lives of 7.04 × 10^8^ and 4.47 × 10^9^ years, respectively. These radionuclides are also chemically toxic. Handling these radionuclides involves a serious risk to human health. Therefore, special precautions with appropriate lab equipment and facilities dedicated to radiation protection are required for handling these radionuclides.

Single crystals of the {U_13_} cluster complex were obtained as a by-product when U^IV^ was dissolved in methanol in the presence of a basic organic ligand. The crystals were obtained from the following two different synthetic routes:

Route A: [UCl(*S*)-PEBA)_3_] (*S*)-PEBA: (*S*,*S*)-*N*,*N*′-bis­(1-phenyl­eth­yl)benzamidinate) was prepared according to a reported procedure (Kloditz *et al.*, 2020 ▸). A solution containing 10 mg of [UCl(*S*)-PEBA)_3_] in 1 mL of methanol was transferred into a quartz cuvette and sealed doubly with a lid and Parafilm in a dry and inert glove box filled with nitro­gen gas. The cuvette was then taken out of the glove box and kept under atmospheric condition. After ten days, dark-black crystals were obtained with a low yield (<1 mg).

Route B: [UCl_2_(salen)_2_(MeOH)_2_] (H_2_salen = *N*,*N*′-bis(salicyl­idene)ethyl­enedi­amine) was prepared according to a reported procedure (Radoske *et al.*, 2020 ▸). A solution containing 7 mg of [UCl_2_(salen)_2_(MeOH)_2_] in 1 mL of methanol was transferred into a quartz cuvette and sealed doubly with a lid and Parafilm in a dry and inert glove box filled with nitro­gen gas. The cuvette was then taken out of the glove box and kept under atmospheric condition. After one week, dark-black crystals were obtained with a low yield (<1 mg).

Synthetic attempts in the absence of an organic ligand did not succeed in obtaining crystals of the {U_13_} polyoxo cluster. It was reported that the reaction between an alcohol mol­ecule (methanol in the present case) and another organic mol­ecule can generate a water mol­ecule, which is the source to trigger the olation/oxolation reaction that could eventually result in the formation of polyoxo clusters (Martin *et al.*, 2018 ▸). Hence, the presence of an organic ligand in an alcohol medium is presumably essential to materialize polyoxo metal cluster complexes. Another possible source of water into the synthetic route is the slow penetration of ambient moisture into the sample cuvette *via* the double sealing, which cannot be completely excluded. Crystals suitable for single crystal X-ray diffraction (SC-XRD) measurements were selected on a polarized light microscope and mounted on a MiTeGen MicroMount^TM^ with mineral oil. Due to the low yield of crystals, additional characterization, such as elemental analysis, FT–IR, powder-XRD, *etc*., was not feasible. Chemicals (except uranium) employed in this study were commercially available from Sigma Aldrich and were used without further purification.

## Refinement   

Crystal data, data collection and structure refinement details are summarized in Table 3 ▸.

All non-hydrogen atoms were refined anisotropically. H atoms of the methoxide groups were placed in the expected geometric positions and treated in a riding mode with *U*_iso_(H) = 1.5 *U*_eq_(C). Three apical ligand positions (Cl1–Cl3) in the asymmetric unit showed pseudo-substitutional disorder between negatively charged methoxide (MeO^−^) and chloride (Cl^−^) ions. This disorder was modelled by constraining the sum of the site occupation factors to unity. Additional constraints (SIMU, DELU and SAME) were applied to avoid chemically unreasonable ellipsoids. Even after the completion of refinement, substantial residual electron density remained around the uranium atoms or within their ionic radii. This is not an uncommon issue in heavy atom structures and was possibly intensified by truncation errors of the Fourier series. Additionally, disorder issues between methoxide and chloride ions caused further residual electron density that could not be modelled in a chemically reasonable manner.

## Supplementary Material

Crystal structure: contains datablock(s) global, I. DOI: 10.1107/S2056989021007623/yz2009sup1.cif

Structure factors: contains datablock(s) I. DOI: 10.1107/S2056989021007623/yz2009Isup2.hkl

Supporting information file. DOI: 10.1107/S2056989021007623/yz2009Isup3.mol

CCDC reference: 2069007

Additional supporting information:  crystallographic information; 3D view; checkCIF report

## Figures and Tables

**Table 1:** Summary of bond-valence-sum (BVS) calculations on the uranium atoms U1–U7 in (I)

Atom	Atomic charge calculated by BVS (v.u.)
U1	3.73
U2	3.99
U3	4.34
U4	3.86
U5	3.77
U6	5.16
U7	3.93
Average	4.11

**Table 2:** Geometrical parameters (Å, °) of cubic uranium polyhedra in different compounds

	U—O distance			O—U—O angle			
Compound	Shortest	Longest	Average	Smallest	Largest	Average	Reference
UO_2_			2.368			70.5	Cooper (1982 ▸)
{U_38_}	2.229	2.520	2.357	68.1	76.5	70.5	Falaize *et al.* (2013*c* ▸)
{U_13_}	2.243	2.290	2.264	69.8	71.2	70.5	This work

**Table 3:** Experimental details

Crystal data	
Chemical formula	[U_13_(CH_3_O)_35.7_Cl_2.3_O_8_]
*M*_r_	4412.01
Crystal system, space group	Triclinic, *P*\overline{1}
Temperature (K)	100
*a*, *b*, *c* (Å)	12.8598 (6), 14.0014 (6), 14.6311 (6)
α, β, γ (°)	117.339 (2), 113.186 (2), 92.373 (2)
*V* (Å^3^)	2069.95 (16)
*Z*	1
Radiation type	Mo *K*α
μ (mm^−1^)	25.48
Crystal size (mm)	0.12 × 0.11 × 0.08

Data collection	
Diffractometer	Bruker D8 Venture
Absorption correction	Multi-scan (Krause *et al.*, 2015 ▸)
*T*_min_, *T*_max_	0.394, 0.746
No. of measured, independent and observed [*I* > 2σ(*I*)] reflections	55319, 7304, 6185
*R*_int_	0.052
(sin θ/λ)_max_ (Å^−1^)	0.595

Refinement	
*R*[*F* ^2^ > 2σ(*F* ^2^)], *wR*(*F* ^2^), *S*	0.038, 0.103, 1.09
No. of reflections	7304
No. of parameters	488
No. of restraints	145
H-atom treatment	H-atom parameters constrained
Δρ_max_, Δρ_min_ (e Å^−3^)	5.02, −3.41

Computer programs: *APEX3* and *SAINT* (Bruker, 2016 ▸), *SHELXT2014/5* (Sheldrick, 2015*a* ▸), *SHELXL2014/7* (Sheldrick, 2015*b* ▸) and *shelXle* (Hübschle *et al.*, 2011 ▸).

## supplementary crystallographic information

### Crystal data

**Table:**

[U_13_(CH_3_O)_35.7_Cl_2.3_O_8_]	*Z* = 1
*M**_r_* = 4412.01	*F*(000) = 1906
Triclinic, *P*1	*D*_x_ = 3.539 Mg m^−^^3^
*a* = 12.8598 (6) Å	Mo *K*α radiation, λ = 0.71073 Å
*b* = 14.0014 (6) Å	Cell parameters from 529 reflections
*c* = 14.6311 (6) Å	θ = 2.2–29.1°
α = 117.339 (2)°	µ = 25.48 mm^−^^1^
β = 113.186 (2)°	*T* = 100 K
γ = 92.373 (2)°	Block, black
*V* = 2069.95 (16) Å^3^	0.12 × 0.11 × 0.08 mm

### Data collection

**Table:**

Bruker D8 Venture diffractometer	6185 reflections with *I* > 2σ(*I*)
Detector resolution: 10.4167 pixels mm^-1^	*R*_int_ = 0.052
generic φ and ο scans	θ_max_ = 25.0°, θ_min_ = 2.7°
Absorption correction: multi-scan (Krause *et al.*, 2015)	*h* = −15→15
*T*_min_ = 0.394, *T*_max_ = 0.746	*k* = −16→16
55319 measured reflections	*l* = −17→17
7304 independent reflections	

### Refinement

**Table:**

Refinement on *F*^2^	Primary atom site location: dual
Least-squares matrix: full	Secondary atom site location: difference Fourier map
*R*[*F*^2^ > 2σ(*F*^2^)] = 0.038	Hydrogen site location: inferred from neighbouring sites
*wR*(*F*^2^) = 0.103	H-atom parameters constrained
*S* = 1.09	*w* = 1/[σ^2^(*F*_o_^2^) + (0.0373*P*)^2^ + 70.429*P*] where *P* = (*F*_o_^2^ + 2*F*_c_^2^)/3
7304 reflections	(Δ/σ)_max_ = 0.001
488 parameters	Δρ_max_ = 5.02 e Å^−^^3^
145 restraints	Δρ_min_ = −3.41 e Å^−^^3^

### Special details

**Table:**

Geometry. All esds (except the esd in the dihedral angle between two l.s. planes) are estimated using the full covariance matrix. The cell esds are taken into account individually in the estimation of esds in distances, angles and torsion angles; correlations between esds in cell parameters are only used when they are defined by crystal symmetry. An approximate (isotropic) treatment of cell esds is used for estimating esds involving l.s. planes.
Refinement. Reflections were merged by SHELXL according to the crystal class for the calculation of statistics and refinement. _reflns_Friedel_fraction is defined as the number of unique Friedel pairs measured divided by the number that would be possible theoretically, ignoring centric projections and systematic absences.

### Fractional atomic coordinates and isotropic or equivalent isotropic displacement parameters (Å^2^)

**Table:**

	*x*	*y*	*z*	*U*_iso_*/*U*_eq_	Occ. (<1)
U1	0.69331 (4)	0.29727 (4)	0.47526 (4)	0.01860 (12)	
Cl1	0.8241 (9)	0.1509 (8)	0.4746 (9)	0.035 (3)	0.43 (2)
O1M	0.785 (2)	0.1771 (16)	0.4625 (19)	0.030 (4)	0.57 (2)
C1M	0.841 (2)	0.098 (2)	0.458 (3)	0.036 (5)	0.57 (2)
H1MA	0.806513	0.050267	0.477592	0.054*	0.57 (2)
H1MB	0.924380	0.132515	0.514146	0.054*	0.57 (2)
H1MC	0.832297	0.051765	0.379736	0.054*	0.57 (2)
U4	0.64636 (4)	0.81258 (4)	0.69281 (4)	0.01947 (12)	
Cl3	0.7477 (10)	1.0349 (10)	0.8252 (9)	0.040 (4)	0.41 (2)
O3M	0.7246 (19)	0.9836 (15)	0.7937 (19)	0.032 (4)	0.59 (2)
C3M	0.781 (3)	1.092 (2)	0.860 (3)	0.049 (7)	0.59 (2)
H3MA	0.845576	1.105852	0.843624	0.074*	0.59 (2)
H3MB	0.813585	1.115315	0.942153	0.074*	0.59 (2)
H3MC	0.726548	1.135521	0.843028	0.074*	0.59 (2)
U5	0.83654 (4)	0.60227 (4)	0.66263 (4)	0.02061 (12)	
Cl2	1.0679 (11)	0.6808 (10)	0.7997 (11)	0.026 (4)	0.33 (2)
O2M	1.0187 (16)	0.6605 (18)	0.7652 (17)	0.031 (4)	0.67 (2)
C2M	1.1339 (19)	0.710 (2)	0.841 (2)	0.038 (5)	0.67 (2)
H2MA	1.158316	0.684020	0.895857	0.056*	0.67 (2)
H2MB	1.147384	0.791002	0.882719	0.056*	0.67 (2)
H2MC	1.179729	0.689926	0.797759	0.056*	0.67 (2)
C1	0.6604 (16)	0.1259 (13)	0.2091 (13)	0.042 (4)	
H1A	0.580311	0.076297	0.157510	0.062*	
H1B	0.708247	0.094877	0.253749	0.062*	
H1C	0.694874	0.132614	0.162608	0.062*	
O1	0.6573 (9)	0.2307 (8)	0.2848 (8)	0.027 (2)	
U2	0.53611 (4)	0.33105 (4)	0.22261 (4)	0.01968 (12)	
C2	0.5492 (17)	0.1741 (14)	−0.0359 (14)	0.047 (4)	
H2A	0.604596	0.221469	−0.038860	0.071*	
H2B	0.471759	0.148096	−0.102995	0.071*	
H2C	0.577377	0.109680	−0.037517	0.071*	
O2	0.5407 (9)	0.2342 (8)	0.0640 (8)	0.034 (2)	
O3	0.3774 (9)	0.3831 (8)	0.1168 (7)	0.028 (2)	
U3	0.33989 (4)	0.53469 (4)	0.24238 (4)	0.01995 (12)	
C3	0.3303 (17)	0.3378 (14)	−0.0051 (12)	0.045 (4)	
H3A	0.259405	0.276313	−0.046428	0.068*	
H3B	0.388827	0.309662	−0.029874	0.068*	
H3C	0.310385	0.396091	−0.022861	0.068*	
C4	0.176 (2)	0.564 (2)	0.0362 (19)	0.072 (7)	
H4A	0.210964	0.567796	−0.011211	0.109*	
H4B	0.152555	0.631791	0.070049	0.109*	
H4C	0.107557	0.498654	−0.012561	0.109*	
O4	0.2354 (11)	0.5561 (10)	0.0995 (11)	0.050 (3)	
C5	0.299 (3)	0.7886 (17)	0.341 (2)	0.079 (7)	
H5A	0.355197	0.797934	0.314725	0.119*	
H5B	0.316529	0.856905	0.414566	0.119*	
H5C	0.218974	0.773996	0.282663	0.119*	
O5	0.3068 (9)	0.6959 (8)	0.3598 (8)	0.031 (2)	
U6	0.500000	0.500000	0.500000	0.01170 (13)	
C6	0.5010 (17)	0.7108 (18)	0.2245 (17)	0.055 (5)	
H6A	0.529374	0.671008	0.167916	0.082*	
H6B	0.552564	0.786931	0.280803	0.082*	
H6C	0.420795	0.713713	0.183990	0.082*	
O6	0.5011 (9)	0.6532 (8)	0.2841 (8)	0.030 (2)	
O8	0.4733 (8)	0.8554 (8)	0.6182 (8)	0.028 (2)	
U7	0.67893 (4)	0.63767 (4)	0.41143 (4)	0.01923 (12)	
C7	0.674 (2)	0.9055 (14)	0.5289 (17)	0.056 (5)	
H7A	0.672716	0.882038	0.454018	0.085*	
H7B	0.746083	0.965425	0.591635	0.085*	
H7C	0.605458	0.932710	0.529703	0.085*	
O7	0.6722 (9)	0.8130 (7)	0.5454 (8)	0.031 (2)	
C8	0.4640 (16)	0.9651 (13)	0.6393 (19)	0.055 (5)	
H8A	0.399752	0.958181	0.570164	0.083*	
H8B	0.537977	1.008320	0.656340	0.083*	
H8C	0.447980	1.003278	0.705612	0.083*	
C9	0.6967 (15)	0.8979 (12)	0.9640 (12)	0.038 (4)	
H9A	0.758341	0.867307	0.996900	0.057*	
H9B	0.649611	0.915329	1.005438	0.057*	
H9C	0.732922	0.966174	0.972065	0.057*	
O9	0.6228 (8)	0.8175 (7)	0.8437 (7)	0.027 (2)	
O10	0.8303 (9)	0.7880 (8)	0.7769 (8)	0.031 (2)	
C10	0.9266 (13)	0.8800 (13)	0.8746 (14)	0.041 (4)	
H10A	1.000438	0.860863	0.880182	0.062*	
H10B	0.921494	0.897387	0.945424	0.062*	
H10C	0.924250	0.945163	0.865598	0.062*	
O11	0.8253 (9)	0.5933 (8)	0.8157 (8)	0.030 (2)	
C11	0.9024 (16)	0.6711 (15)	0.9356 (13)	0.048 (4)	
H11A	0.974037	0.708951	0.943703	0.072*	
H11B	0.922856	0.631307	0.977792	0.072*	
H11C	0.863204	0.726483	0.967794	0.072*	
O12	0.8746 (9)	0.4274 (8)	0.6120 (9)	0.032 (2)	
C12	0.9709 (14)	0.4066 (14)	0.6837 (16)	0.051 (5)	
H12A	0.974763	0.437853	0.760510	0.077*	
H12B	1.043906	0.441831	0.691664	0.077*	
H12C	0.960644	0.325887	0.648404	0.077*	
O13	0.8658 (9)	0.6767 (8)	0.5580 (9)	0.031 (2)	
C13	0.9615 (15)	0.7636 (15)	0.6003 (15)	0.046 (4)	
H13A	0.963114	0.766412	0.535197	0.069*	
H13B	1.034609	0.750442	0.642451	0.069*	
H13C	0.953693	0.834756	0.653022	0.069*	
O14	0.7675 (11)	0.7281 (10)	0.3648 (9)	0.045 (3)	
C14	0.8158 (16)	0.7802 (17)	0.3389 (18)	0.062 (6)	
H14A	0.834364	0.728577	0.278184	0.093*	
H14B	0.888592	0.834229	0.407567	0.093*	
H14C	0.763666	0.819879	0.310097	0.093*	
O15	0.6499 (9)	0.4803 (8)	0.2400 (8)	0.027 (2)	
C15	0.6596 (19)	0.4833 (15)	0.1523 (15)	0.054 (5)	
H15A	0.689802	0.421427	0.114854	0.082*	
H15B	0.713903	0.554408	0.183615	0.082*	
H15C	0.582226	0.476164	0.095123	0.082*	
O16	0.7331 (8)	0.4585 (8)	0.4341 (8)	0.027 (2)	
C16	0.8348 (19)	0.4483 (16)	0.4207 (16)	0.063 (6)	
H16A	0.831414	0.369029	0.378368	0.094*	
H16B	0.904029	0.486089	0.497337	0.094*	
H16C	0.840162	0.482610	0.376880	0.094*	
O17	0.6641 (8)	0.4672 (7)	0.5997 (7)	0.0200 (18)	
O18	0.5061 (8)	0.5022 (7)	0.3469 (7)	0.0191 (18)	
O19	0.4861 (7)	0.6687 (7)	0.6212 (7)	0.0168 (17)	
O20	0.6572 (7)	0.6365 (7)	0.5677 (7)	0.0176 (17)	

### Atomic displacement parameters (Å^2^)

**Table:**

	*U*^11^	*U*^22^	*U*^33^	*U*^12^	*U*^13^	*U*^23^
U1	0.0266 (3)	0.0149 (2)	0.0179 (2)	0.00949 (19)	0.0113 (2)	0.01012 (19)
Cl1	0.035 (6)	0.028 (6)	0.045 (5)	0.017 (4)	0.019 (4)	0.020 (4)
O1M	0.033 (7)	0.028 (7)	0.032 (6)	0.018 (5)	0.016 (6)	0.016 (6)
C1M	0.032 (10)	0.034 (11)	0.052 (11)	0.020 (8)	0.030 (9)	0.019 (10)
U4	0.0294 (3)	0.0112 (2)	0.0149 (2)	0.00420 (19)	0.0090 (2)	0.00584 (18)
Cl3	0.050 (7)	0.023 (7)	0.023 (7)	−0.004 (6)	0.012 (5)	0.000 (5)
O3M	0.036 (7)	0.015 (6)	0.019 (7)	−0.002 (7)	0.005 (6)	−0.001 (6)
C3M	0.047 (13)	0.021 (9)	0.032 (12)	−0.008 (11)	0.003 (10)	−0.007 (11)
U5	0.0247 (3)	0.0175 (2)	0.0164 (2)	0.00476 (19)	0.0066 (2)	0.00890 (19)
Cl2	0.011 (7)	0.028 (6)	0.024 (7)	−0.007 (6)	0.003 (5)	0.007 (5)
O2M	0.022 (6)	0.032 (7)	0.024 (7)	−0.002 (6)	0.004 (6)	0.010 (5)
C2M	0.013 (7)	0.052 (11)	0.034 (11)	−0.002 (9)	0.013 (7)	0.011 (9)
C1	0.065 (11)	0.034 (9)	0.027 (8)	0.027 (8)	0.026 (8)	0.012 (7)
O1	0.043 (6)	0.024 (5)	0.029 (5)	0.020 (4)	0.025 (5)	0.016 (4)
U2	0.0328 (3)	0.0153 (2)	0.0148 (2)	0.0093 (2)	0.0140 (2)	0.00798 (19)
C2	0.076 (13)	0.035 (9)	0.041 (9)	0.026 (9)	0.046 (10)	0.010 (8)
O2	0.048 (6)	0.029 (5)	0.030 (5)	0.011 (5)	0.020 (5)	0.017 (5)
O3	0.045 (6)	0.026 (5)	0.013 (4)	0.012 (4)	0.013 (4)	0.010 (4)
U3	0.0284 (3)	0.0195 (2)	0.0147 (2)	0.0087 (2)	0.0088 (2)	0.01164 (19)
C3	0.070 (12)	0.045 (10)	0.012 (7)	0.019 (9)	0.012 (7)	0.014 (7)
C4	0.089 (17)	0.075 (15)	0.051 (12)	−0.002 (13)	0.026 (11)	0.039 (12)
O4	0.052 (8)	0.042 (7)	0.040 (6)	0.006 (6)	0.023 (6)	0.009 (5)
C5	0.17 (2)	0.056 (11)	0.081 (14)	0.063 (14)	0.092 (16)	0.056 (11)
O5	0.051 (6)	0.026 (5)	0.028 (5)	0.017 (5)	0.022 (5)	0.020 (4)
U6	0.0178 (3)	0.0086 (3)	0.0095 (3)	0.0054 (2)	0.0062 (2)	0.0053 (2)
C6	0.051 (11)	0.076 (14)	0.065 (12)	0.019 (10)	0.024 (10)	0.058 (12)
O6	0.044 (6)	0.029 (5)	0.032 (5)	0.015 (5)	0.020 (5)	0.024 (5)
O8	0.034 (5)	0.023 (5)	0.032 (5)	0.010 (4)	0.014 (5)	0.018 (4)
U7	0.0278 (3)	0.0162 (2)	0.0172 (2)	0.00635 (19)	0.0118 (2)	0.01013 (19)
C7	0.103 (16)	0.037 (10)	0.058 (11)	0.025 (10)	0.046 (12)	0.038 (9)
O7	0.057 (7)	0.011 (4)	0.030 (5)	0.012 (4)	0.024 (5)	0.013 (4)
C8	0.047 (10)	0.023 (8)	0.085 (14)	0.007 (7)	0.016 (10)	0.031 (9)
C9	0.053 (10)	0.023 (7)	0.015 (7)	−0.010 (7)	0.011 (7)	−0.001 (6)
O9	0.037 (6)	0.018 (5)	0.015 (4)	0.002 (4)	0.006 (4)	0.006 (4)
O10	0.037 (6)	0.022 (5)	0.014 (4)	−0.005 (4)	0.005 (4)	0.001 (4)
C10	0.030 (8)	0.036 (9)	0.044 (9)	−0.006 (7)	0.012 (7)	0.016 (8)
O11	0.038 (6)	0.029 (5)	0.014 (4)	0.009 (4)	0.006 (4)	0.010 (4)
C11	0.048 (10)	0.047 (10)	0.027 (8)	−0.003 (8)	0.009 (8)	0.011 (8)
O12	0.032 (5)	0.022 (5)	0.037 (6)	0.012 (4)	0.009 (5)	0.017 (5)
C12	0.031 (9)	0.037 (9)	0.050 (10)	0.014 (7)	0.000 (8)	0.013 (8)
O13	0.033 (5)	0.029 (5)	0.038 (6)	0.005 (4)	0.016 (5)	0.022 (5)
C13	0.044 (10)	0.060 (11)	0.047 (10)	0.004 (8)	0.026 (8)	0.033 (9)
O14	0.055 (8)	0.043 (7)	0.027 (6)	0.021 (6)	0.015 (6)	0.013 (5)
C14	0.034 (10)	0.058 (13)	0.055 (12)	0.008 (9)	0.015 (9)	0.004 (10)
O15	0.045 (6)	0.024 (5)	0.022 (5)	0.012 (4)	0.023 (5)	0.013 (4)
C15	0.080 (14)	0.036 (9)	0.045 (10)	−0.006 (9)	0.040 (10)	0.011 (8)
O16	0.030 (5)	0.030 (5)	0.030 (5)	0.012 (4)	0.019 (4)	0.016 (4)
C16	0.086 (16)	0.047 (12)	0.042 (11)	0.002 (11)	0.030 (11)	0.015 (9)
O17	0.027 (5)	0.020 (4)	0.015 (4)	0.007 (4)	0.007 (4)	0.014 (4)
O18	0.029 (5)	0.016 (4)	0.014 (4)	0.011 (4)	0.010 (4)	0.009 (4)
O19	0.026 (5)	0.011 (4)	0.014 (4)	0.007 (3)	0.010 (4)	0.007 (3)
O20	0.022 (4)	0.017 (4)	0.017 (4)	0.008 (4)	0.008 (4)	0.012 (4)

### Geometric parameters (Å, º)

**Table:**

U1—O1M	2.076 (19)	C4—H4B	0.9800
U1—O12	2.320 (10)	C4—H4C	0.9800
U1—O1	2.326 (9)	C5—O5	1.441 (19)
U1—O5^i^	2.371 (9)	C5—H5A	0.9800
U1—O8^i^	2.378 (9)	C5—H5B	0.9800
U1—O19^i^	2.385 (8)	C5—H5C	0.9800
U1—O17	2.399 (9)	U6—O17^i^	2.249 (8)
U1—O16	2.668 (9)	U6—O17	2.249 (8)
U1—Cl1	2.704 (11)	U6—O20^i^	2.255 (8)
O1M—C1M	1.34 (3)	U6—O20	2.255 (8)
C1M—H1MA	0.9800	U6—O19^i^	2.279 (8)
C1M—H1MB	0.9800	U6—O19	2.279 (8)
C1M—H1MC	0.9800	U6—O18^i^	2.287 (8)
U4—O3M	2.076 (18)	U6—O18	2.287 (8)
U4—O8	2.303 (9)	C6—O6	1.432 (17)
U4—O7	2.310 (9)	C6—H6A	0.9800
U4—O9	2.315 (9)	C6—H6B	0.9800
U4—O10	2.322 (10)	C6—H6C	0.9800
U4—O19	2.328 (8)	O6—U7	2.410 (10)
U4—O20	2.352 (8)	O8—C8	1.442 (17)
U4—Cl3	2.698 (11)	U7—O14	2.157 (14)
O3M—C3M	1.34 (3)	U7—O15	2.324 (9)
C3M—H3MA	0.9800	U7—O13	2.330 (10)
C3M—H3MB	0.9800	U7—O7	2.368 (9)
C3M—H3MC	0.9800	U7—O18	2.404 (9)
U5—O2M	2.086 (18)	U7—O20	2.417 (8)
U5—O13	2.337 (9)	U7—O16	2.759 (9)
U5—O12	2.346 (9)	C7—O7	1.423 (17)
U5—O11	2.359 (9)	C7—H7A	0.9800
U5—O20	2.379 (8)	C7—H7B	0.9800
U5—O10	2.387 (10)	C7—H7C	0.9800
U5—O17	2.406 (9)	C8—H8A	0.9800
U5—Cl2	2.662 (12)	C8—H8B	0.9800
U5—O16	2.664 (9)	C8—H8C	0.9800
O2M—C2M	1.35 (3)	C9—O9	1.429 (16)
C2M—H2MA	0.9800	C9—H9A	0.9800
C2M—H2MB	0.9800	C9—H9B	0.9800
C2M—H2MC	0.9800	C9—H9C	0.9800
C1—O1	1.389 (16)	O10—C10	1.420 (17)
C1—H1A	0.9800	C10—H10A	0.9800
C1—H1B	0.9800	C10—H10B	0.9800
C1—H1C	0.9800	C10—H10C	0.9800
O1—U2	2.339 (9)	O11—C11	1.426 (18)
U2—O2	2.110 (10)	C11—H11A	0.9800
U2—O15	2.353 (9)	C11—H11B	0.9800
U2—O9^i^	2.383 (9)	C11—H11C	0.9800
U2—O3	2.401 (9)	O12—C12	1.416 (18)
U2—O19^i^	2.411 (8)	C12—H12A	0.9800
U2—O18	2.413 (8)	C12—H12B	0.9800
U2—O16	2.749 (10)	C12—H12C	0.9800
C2—O2	1.363 (17)	O13—C13	1.400 (18)
C2—H2A	0.9800	C13—H13A	0.9800
C2—H2B	0.9800	C13—H13B	0.9800
C2—H2C	0.9800	C13—H13C	0.9800
O3—C3	1.425 (15)	O14—C14	1.21 (2)
O3—U3	2.305 (9)	C14—H14A	0.9800
U3—O4	2.160 (14)	C14—H14B	0.9800
U3—O6	2.284 (10)	C14—H14C	0.9800
U3—O18	2.293 (8)	O15—C15	1.356 (18)
U3—O11^i^	2.305 (10)	C15—H15A	0.9800
U3—O5	2.307 (9)	C15—H15B	0.9800
U3—O17^i^	2.344 (8)	C15—H15C	0.9800
C3—H3A	0.9800	O16—C16	1.40 (2)
C3—H3B	0.9800	C16—H16A	0.9800
C3—H3C	0.9800	C16—H16B	0.9800
C4—O4	0.99 (2)	C16—H16C	0.9800
C4—H4A	0.9800		
			
O1M—U1—O12	86.1 (7)	C4—O4—U3	170.5 (18)
O1M—U1—O1	83.1 (7)	O5—C5—H5A	109.5
O12—U1—O1	113.1 (4)	O5—C5—H5B	109.5
O12—U1—O5^i^	83.0 (4)	H5A—C5—H5B	109.5
O1—U1—O5^i^	161.1 (3)	O5—C5—H5C	109.5
O12—U1—O8^i^	160.6 (3)	H5A—C5—H5C	109.5
O1—U1—O8^i^	82.2 (3)	H5B—C5—H5C	109.5
O5^i^—U1—O8^i^	80.1 (3)	C5—O5—U3	123.3 (9)
O12—U1—O19^i^	127.3 (3)	C5—O5—U1^i^	122.8 (9)
O1—U1—O19^i^	72.2 (3)	U3—O5—U1^i^	113.6 (4)
O5^i^—U1—O19^i^	106.7 (3)	O17^i^—U6—O17	180.0 (5)
O8^i^—U1—O19^i^	67.4 (3)	O17^i^—U6—O20^i^	71.0 (3)
O1M—U1—O17	147.4 (6)	O17—U6—O20^i^	109.0 (3)
O12—U1—O17	71.0 (3)	O17^i^—U6—O20	109.0 (3)
O1—U1—O17	126.8 (3)	O17—U6—O20	71.0 (3)
O5^i^—U1—O17	66.5 (3)	O20^i^—U6—O20	180.0
O8^i^—U1—O17	110.3 (3)	O17^i^—U6—O19^i^	108.9 (3)
O19^i^—U1—O17	66.8 (3)	O17—U6—O19^i^	71.1 (3)
O1M—U1—O16	124.2 (7)	O20^i^—U6—O19^i^	70.0 (3)
O12—U1—O16	67.0 (3)	O20—U6—O19^i^	110.0 (3)
O1—U1—O16	66.4 (3)	O17^i^—U6—O19	71.1 (3)
O5^i^—U1—O16	131.6 (3)	O17—U6—O19	108.9 (3)
O8^i^—U1—O16	132.1 (3)	O20^i^—U6—O19	110.0 (3)
O19^i^—U1—O16	69.0 (3)	O20—U6—O19	70.0 (3)
O17—U1—O16	68.1 (3)	O19^i^—U6—O19	180.0
O12—U1—Cl1	82.5 (3)	O17^i^—U6—O18^i^	110.4 (3)
O1—U1—Cl1	86.4 (3)	O17—U6—O18^i^	69.6 (3)
O5^i^—U1—Cl1	86.0 (3)	O20^i^—U6—O18^i^	70.8 (3)
O8^i^—U1—Cl1	86.8 (3)	O20—U6—O18^i^	109.2 (3)
O19^i^—U1—Cl1	148.1 (3)	O19^i^—U6—O18^i^	109.3 (3)
O17—U1—Cl1	143.4 (3)	O19—U6—O18^i^	70.7 (3)
O16—U1—Cl1	124.0 (3)	O17^i^—U6—O18	69.6 (3)
C1M—O1M—U1	176 (2)	O17—U6—O18	110.4 (3)
O1M—C1M—H1MA	109.5	O20^i^—U6—O18	109.2 (3)
O1M—C1M—H1MB	109.5	O20—U6—O18	70.8 (3)
H1MA—C1M—H1MB	109.5	O19^i^—U6—O18	70.7 (3)
O1M—C1M—H1MC	109.5	O19—U6—O18	109.3 (3)
H1MA—C1M—H1MC	109.5	O18^i^—U6—O18	180.0
H1MB—C1M—H1MC	109.5	O6—C6—H6A	109.5
O3M—U4—O8	86.4 (7)	O6—C6—H6B	109.5
O3M—U4—O7	88.1 (7)	H6A—C6—H6B	109.5
O8—U4—O7	85.1 (3)	O6—C6—H6C	109.5
O3M—U4—O9	90.3 (7)	H6A—C6—H6C	109.5
O8—U4—O9	94.8 (3)	H6B—C6—H6C	109.5
O7—U4—O9	178.4 (3)	C6—O6—U3	125.1 (10)
O3M—U4—O10	87.9 (7)	C6—O6—U7	122.8 (10)
O8—U4—O10	174.3 (3)	U3—O6—U7	110.7 (3)
O7—U4—O10	93.9 (4)	C8—O8—U4	124.9 (9)
O9—U4—O10	86.1 (3)	C8—O8—U1^i^	122.9 (9)
O3M—U4—O19	146.4 (7)	U4—O8—U1^i^	111.7 (4)
O8—U4—O19	69.6 (3)	O14—U7—O15	85.3 (4)
O7—U4—O19	112.0 (3)	O14—U7—O13	85.2 (4)
O9—U4—O19	69.4 (3)	O15—U7—O13	109.0 (3)
O10—U4—O19	115.9 (3)	O14—U7—O7	86.7 (4)
O3M—U4—O20	146.0 (7)	O15—U7—O7	162.5 (3)
O8—U4—O20	115.1 (3)	O13—U7—O7	85.8 (4)
O7—U4—O20	69.1 (3)	O14—U7—O18	146.0 (3)
O9—U4—O20	112.3 (3)	O15—U7—O18	72.6 (3)
O10—U4—O20	69.5 (3)	O13—U7—O18	126.0 (3)
O19—U4—O20	67.6 (3)	O7—U7—O18	106.7 (3)
O8—U4—Cl3	86.4 (3)	O14—U7—O6	85.3 (4)
O7—U4—Cl3	88.5 (3)	O15—U7—O6	83.1 (3)
O9—U4—Cl3	89.9 (3)	O13—U7—O6	163.9 (3)
O10—U4—Cl3	88.0 (3)	O7—U7—O6	80.7 (3)
O19—U4—Cl3	146.1 (3)	O18—U7—O6	67.0 (3)
O20—U4—Cl3	146.3 (3)	O14—U7—O20	145.9 (3)
C3M—O3M—U4	177 (3)	O15—U7—O20	125.5 (3)
O3M—C3M—H3MA	109.5	O13—U7—O20	72.0 (3)
O3M—C3M—H3MB	109.5	O7—U7—O20	67.1 (3)
H3MA—C3M—H3MB	109.5	O18—U7—O20	66.2 (3)
O3M—C3M—H3MC	109.5	O6—U7—O20	110.2 (3)
H3MA—C3M—H3MC	109.5	O14—U7—O16	125.7 (4)
H3MB—C3M—H3MC	109.5	O15—U7—O16	65.7 (3)
O2M—U5—O13	85.7 (7)	O13—U7—O16	64.9 (3)
O2M—U5—O12	82.7 (6)	O7—U7—O16	131.1 (3)
O13—U5—O12	113.4 (4)	O18—U7—O16	68.1 (3)
O2M—U5—O11	87.6 (7)	O6—U7—O16	131.1 (3)
O13—U5—O11	160.2 (3)	O20—U7—O16	66.9 (3)
O12—U5—O11	84.1 (3)	O7—C7—H7A	109.5
O2M—U5—O20	148.5 (6)	O7—C7—H7B	109.5
O13—U5—O20	72.5 (3)	H7A—C7—H7B	109.5
O12—U5—O20	126.5 (3)	O7—C7—H7C	109.5
O11—U5—O20	105.1 (3)	H7A—C7—H7C	109.5
O2M—U5—O10	86.8 (6)	H7B—C7—H7C	109.5
O13—U5—O10	81.6 (3)	C7—O7—U4	124.4 (9)
O12—U5—O10	160.8 (3)	C7—O7—U7	122.0 (9)
O11—U5—O10	79.4 (3)	U4—O7—U7	113.5 (3)
O20—U5—O10	68.0 (3)	O8—C8—H8A	109.5
O2M—U5—O17	143.9 (7)	O8—C8—H8B	109.5
O13—U5—O17	126.7 (3)	H8A—C8—H8B	109.5
O12—U5—O17	70.4 (3)	O8—C8—H8C	109.5
O11—U5—O17	66.5 (3)	H8A—C8—H8C	109.5
O20—U5—O17	66.3 (3)	H8B—C8—H8C	109.5
O10—U5—O17	111.1 (3)	O9—C9—H9A	109.5
O13—U5—Cl2	86.7 (4)	O9—C9—H9B	109.5
O12—U5—Cl2	83.9 (4)	H9A—C9—H9B	109.5
O11—U5—Cl2	86.2 (4)	O9—C9—H9C	109.5
O20—U5—Cl2	147.9 (4)	H9A—C9—H9C	109.5
O10—U5—Cl2	85.3 (4)	H9B—C9—H9C	109.5
O17—U5—Cl2	143.6 (4)	C9—O9—U4	124.5 (8)
O2M—U5—O16	123.0 (6)	C9—O9—U2^i^	123.0 (8)
O13—U5—O16	66.4 (3)	U4—O9—U2^i^	112.5 (3)
O12—U5—O16	66.7 (3)	C10—O10—U4	121.1 (9)
O11—U5—O16	132.0 (3)	C10—O10—U5	127.1 (9)
O20—U5—O16	69.0 (3)	U4—O10—U5	111.2 (4)
O10—U5—O16	132.2 (3)	O10—C10—H10A	109.5
O17—U5—O16	68.1 (3)	O10—C10—H10B	109.5
Cl2—U5—O16	124.9 (3)	H10A—C10—H10B	109.5
C2M—O2M—U5	173 (2)	O10—C10—H10C	109.5
O2M—C2M—H2MA	109.5	H10A—C10—H10C	109.5
O2M—C2M—H2MB	109.5	H10B—C10—H10C	109.5
H2MA—C2M—H2MB	109.5	C11—O11—U3^i^	121.4 (9)
O2M—C2M—H2MC	109.5	C11—O11—U5	123.7 (9)
H2MA—C2M—H2MC	109.5	U3^i^—O11—U5	114.2 (4)
H2MB—C2M—H2MC	109.5	O11—C11—H11A	109.5
O1—C1—H1A	109.5	O11—C11—H11B	109.5
O1—C1—H1B	109.5	H11A—C11—H11B	109.5
H1A—C1—H1B	109.5	O11—C11—H11C	109.5
O1—C1—H1C	109.5	H11A—C11—H11C	109.5
H1A—C1—H1C	109.5	H11B—C11—H11C	109.5
H1B—C1—H1C	109.5	C12—O12—U1	124.1 (9)
C1—O1—U1	126.0 (8)	C12—O12—U5	125.1 (9)
C1—O1—U2	123.2 (9)	U1—O12—U5	105.3 (4)
U1—O1—U2	105.4 (3)	O12—C12—H12A	109.5
O2—U2—O1	84.8 (3)	O12—C12—H12B	109.5
O2—U2—O15	84.7 (3)	H12A—C12—H12B	109.5
O1—U2—O15	109.8 (3)	O12—C12—H12C	109.5
O2—U2—O9^i^	88.0 (3)	H12A—C12—H12C	109.5
O1—U2—O9^i^	85.5 (3)	H12B—C12—H12C	109.5
O15—U2—O9^i^	162.4 (3)	C13—O13—U7	124.0 (9)
O2—U2—O3	86.0 (3)	C13—O13—U5	125.9 (9)
O1—U2—O3	164.1 (3)	U7—O13—U5	105.2 (4)
O15—U2—O3	82.2 (3)	O13—C13—H13A	109.5
O9^i^—U2—O3	81.3 (3)	O13—C13—H13B	109.5
O2—U2—O19^i^	146.2 (3)	H13A—C13—H13B	109.5
O1—U2—O19^i^	71.5 (3)	O13—C13—H13C	109.5
O15—U2—O19^i^	125.4 (3)	H13A—C13—H13C	109.5
O9^i^—U2—O19^i^	66.9 (3)	H13B—C13—H13C	109.5
O3—U2—O19^i^	110.7 (3)	C14—O14—U7	178.8 (13)
O2—U2—O18	146.1 (3)	O14—C14—H14A	109.5
O1—U2—O18	125.7 (3)	O14—C14—H14B	109.5
O15—U2—O18	71.9 (3)	H14A—C14—H14B	109.5
O9^i^—U2—O18	106.9 (3)	O14—C14—H14C	109.5
O3—U2—O18	67.1 (3)	H14A—C14—H14C	109.5
O19^i^—U2—O18	66.4 (3)	H14B—C14—H14C	109.5
O2—U2—O16	124.0 (3)	C15—O15—U7	124.3 (9)
O1—U2—O16	64.9 (3)	C15—O15—U2	126.2 (9)
O15—U2—O16	65.5 (3)	U7—O15—U2	105.7 (3)
O9^i^—U2—O16	131.2 (3)	O15—C15—H15A	109.5
O3—U2—O16	130.9 (3)	O15—C15—H15B	109.5
O19^i^—U2—O16	67.2 (3)	H15A—C15—H15B	109.5
O18—U2—O16	68.2 (3)	O15—C15—H15C	109.5
O2—C2—H2A	109.5	H15A—C15—H15C	109.5
O2—C2—H2B	109.5	H15B—C15—H15C	109.5
H2A—C2—H2B	109.5	C16—O16—U5	98.1 (9)
O2—C2—H2C	109.5	C16—O16—U1	103.3 (10)
H2A—C2—H2C	109.5	U5—O16—U1	88.2 (3)
H2B—C2—H2C	109.5	C16—O16—U2	110.3 (9)
C2—O2—U2	177.3 (11)	U5—O16—U2	151.6 (4)
C3—O3—U3	125.8 (9)	U1—O16—U2	86.5 (3)
C3—O3—U2	122.6 (9)	C16—O16—U7	105.4 (10)
U3—O3—U2	110.7 (3)	U5—O16—U7	86.3 (3)
O4—U3—O6	89.5 (4)	U1—O16—U7	151.3 (4)
O4—U3—O18	151.1 (4)	U2—O16—U7	85.2 (3)
O6—U3—O18	71.0 (3)	O16—C16—H16A	109.5
O4—U3—O11^i^	87.5 (4)	O16—C16—H16B	109.5
O6—U3—O11^i^	175.1 (3)	H16A—C16—H16B	109.5
O18—U3—O11^i^	110.3 (3)	O16—C16—H16C	109.5
O4—U3—O3	89.2 (4)	H16A—C16—H16C	109.5
O6—U3—O3	91.3 (3)	H16B—C16—H16C	109.5
O18—U3—O3	70.7 (3)	U6—O17—U3^i^	111.0 (4)
O11^i^—U3—O3	84.9 (3)	U6—O17—U1	111.2 (3)
O4—U3—O5	86.8 (4)	U3^i^—O17—U1	111.3 (3)
O6—U3—O5	84.5 (4)	U6—O17—U5	110.8 (3)
O18—U3—O5	111.4 (3)	U3^i^—O17—U5	111.0 (3)
O11^i^—U3—O5	99.2 (4)	U1—O17—U5	101.1 (3)
O3—U3—O5	174.2 (3)	U6—O18—U3	111.5 (4)
O4—U3—O17^i^	141.1 (4)	U6—O18—U7	111.1 (3)
O6—U3—O17^i^	116.2 (3)	U3—O18—U7	110.6 (3)
O18—U3—O17^i^	67.9 (3)	U6—O18—U2	111.2 (3)
O11^i^—U3—O17^i^	68.3 (3)	U3—O18—U2	110.7 (3)
O3—U3—O17^i^	117.1 (3)	U7—O18—U2	101.4 (3)
O5—U3—O17^i^	68.5 (3)	U6—O19—U4	111.2 (3)
O3—C3—H3A	109.5	U6—O19—U1^i^	110.7 (3)
O3—C3—H3B	109.5	U4—O19—U1^i^	110.6 (3)
H3A—C3—H3B	109.5	U6—O19—U2^i^	111.5 (3)
O3—C3—H3C	109.5	U4—O19—U2^i^	111.0 (3)
H3A—C3—H3C	109.5	U1^i^—O19—U2^i^	101.4 (3)
H3B—C3—H3C	109.5	U6—O20—U4	111.2 (3)
O4—C4—H4A	109.5	U6—O20—U5	111.6 (3)
O4—C4—H4B	109.5	U4—O20—U5	110.5 (3)
H4A—C4—H4B	109.5	U6—O20—U7	111.7 (3)
O4—C4—H4C	109.5	U4—O20—U7	110.2 (3)
H4A—C4—H4C	109.5	U5—O20—U7	101.3 (3)
H4B—C4—H4C	109.5		

Symmetry code: (i) −*x*+1, −*y*+1, −*z*+1.
